# Binge Drinking and Risk of Breast Cancer: Results from the SUN (‘Seguimiento Universidad de Navarra’) Project

**DOI:** 10.3390/nu12030731

**Published:** 2020-03-10

**Authors:** Rodrigo Sánchez-Bayona, Alfredo Gea, Itziar Gardeazabal, Andrea Romanos-Nanclares, Miguel Ángel Martínez-González, Maira Bes-Rastrollo, Marta Santisteban, Estefanía Toledo

**Affiliations:** 1 Department of Preventive Medicine and Public Health, School of Medicine, University of Navarra, 31008 Pamplona, Spain; rsanchez.1@unav.es (R.S.-B.); gardeitzi@gmail.com (I.G.); aromanos@alumni.unav.es (A.R.-N.); mamartinez@unav.es (M.Á.M.-G.); mbes@unav.es (M.B.-R.); etoledo@unav.es (E.T.); 2 Department of Clinical Oncology, Clínica Universidad de Navarra, 31008 Pamplona, Spain; msantisteb@unav.es; 3 Centro de Investigación Biomédica en Red Área de Fisiología de la Obesidad y la Nutrición (CIBEROBN), 28029 Madrid, Spain; 4 IdiSNA, Navarra Institute for Health Research, 31008 Pamplona, Spain; 5 Department of Clinical Oncology, Hospital Universitario Marqués de Valdecilla, 39008 Santander, Spain; 6 Harvard T.H. Chan School of Public Health, Boston, MA 02115, USA

**Keywords:** breast cancer, alcohol, binge drinking, cohort

## Abstract

Alcohol intake is associated with the risk of breast cancer. Different patterns of alcohol-drinking may have different effects on breast cancer even when keeping constant the total amount of alcohol consumed. We aimed to assess the association between binge drinking and breast cancer risk. The SUN Project is a Spanish dynamic prospective cohort of university graduates initiated in 1999. In the 556-item lifestyle baseline questionnaire a validated food-frequency questionnaire was embedded. Participants completed biennial follow-up questionnaires. Cox regression models were used to estimate the hazard ratio (HR) for breast cancer associated with the exposure to binge drinking. A stratified analysis was performed according to menopausal status. We included 9577 women (mean age = 34 years, SD = 10 years), with a median follow-up of 11.8 years. Among 104,932 women-years of follow-up, we confirmed 88 incident cases of breast cancer. Women in the binge drinking group showed a higher risk of breast cancer (HR = 1.76; 95% CI: 1.03–2.99) compared to women in the non-binge drinking category. In the stratified analysis, a 2-fold higher risk for premenopausal breast cancer was associated with binge drinking habit (HR = 2.06; 95% CI: 1.11–3.82). This study adds new evidence on the association of binge drinking with breast cancer risk.

## 1. Introduction

In recent years, the prevalence of heavy drinking and binge drinking in developed countries has increased notably [1]. Reportedly, almost 70% of youth alcohol consumption adopts the pattern of binge drinking, defined as consuming four or more alcoholic drinks on one occasion (5 for men) [2]. It is also defined as a pattern of drinking that leads to blood alcohol concentrations of 0.08 g/dL [3], or intake of ≥60 g of pure alcohol on at least one occasion in the past 30 days [4]. In contrast to this drinking pattern, in the Mediterranean countries, alcohol intake is traditionally moderate, spread out over the week, preferably from wine and consumed with meals and without excess [5]. This Mediterranean alcohol-drinking pattern has been previously explored in the SUN cohort and it was associated with lower all-cause mortality than other drinking patterns providing having the same amount of alcohol [6]. Binge drinking, opposite to customary Mediterranean drinking habits, might be a consequence of the current westernization of the Mediterranean countries.

In 2010, the International Agency for Research on Cancer (IARC) classified alcohol as a Group 1 carcinogen [7]. Epidemiologic studies focused on breast cancer risk generally have assessed an average amount of alcohol intake in a specified time or period, but they have not usually accounted for the effect of a heavy amount of alcohol consumed on a single drinking occasion. This binge drinking pattern normally results in higher alcohol levels in the blood than having a single drink at one time, which has been associated with a variety of adverse health outcomes (e.g., mental disorders, myocardial infarction, stroke) [8]. Alcohol consumption has been consistently associated with a higher risk of breast cancer in women. It has been estimated that, for each 10 g (~1 drink) of alcohol consumed daily by adult women, the risk of breast cancer increases by 7–10% [9]. A dose-response meta-analysis of prospective cohort studies found a 10% (RR = 1.10, 95% CI = 1.08–1.13) relative increase in breast cancer risk with every 10 g per day of total alcohol intake [10]. Furthermore, this association was observed in both premenopausal and postmenopausal women.

Previous observational prospective studies found a 30–50% relative increase in breast cancer risk associated with binge drinking [9,11,12]. However, the definition of binge varied across the studies, and the choice of the comparison group was not consistent among them.

Some experimental studies have linked binge drinking with an increase in inflammation levels as well as insulin resistance [13]. Both mechanisms are hypothesized to play a role in the development of breast cancer [14]. Notwithstanding, little is known about the impact of binge drinking on breast cancer risk.

We aimed to assess the impact of episodic heavy drinking and the subsequent risk of breast cancer incidence in a middle-aged Mediterranean cohort of university graduates.

## 2. Materials and Methods

### 2.1. Study Population

The ‘Seguimiento Universidad de Navarra’ (University of Navarra Follow-up) (SUN) Project is a dynamic and multi-purpose cohort study entirely composed of university graduates. Recruitment started in 1999 and is currently ongoing. Participants are invited to complete a 556-item baseline questionnaire that includes questions about lifestyle, sociodemographic, anthropometric and medical variables. Those who completed the baseline questionnaire are contacted biennially thereafter and inquired about changes in their lifestyles and incident diseases. Details of the design and methods of this cohort study have been described elsewhere [15].

**Figure 1:**
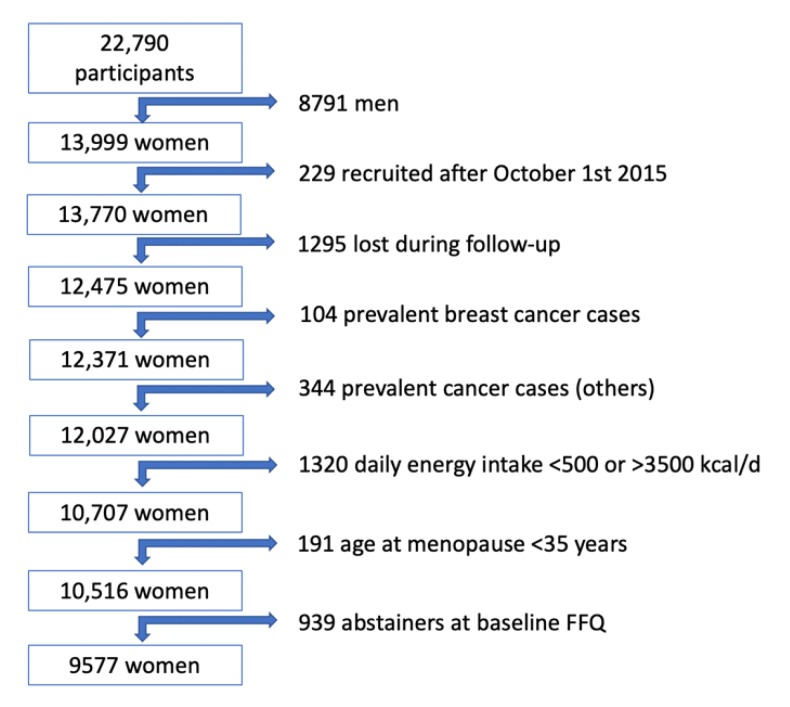
Flowchart of participants in the SUN Project, 1999–2018.

Up to December 2018, 22,790 participants answered the baseline questionnaire. For the present analysis, 8791 men were excluded. We also excluded 229 participants who answered the baseline questionnaire after October 1st, 2015 to ensure a follow-up period of at least 2 years, and 1295 participants due to lack of follow-up (overall retention 91%). We further excluded 104 women who reported a previous breast cancer, and we also excluded participants with prevalent or previous diagnosis of cancer (other than breast; *n* = 344) in the baseline questionnaire. We also excluded 1320 women with a daily energy intake below 500 or above 3500 kcal/d [16] and 191 women reporting an age at menopause younger than 35 years. Participants classified as abstainers at baseline food-frequency questionnaire (FFQ) were also excluded (939 participants) to avoid the potential sick-quitter effect and to compare the drinking pattern (binge or not) only among drinkers. For our analyses, 9577 women were finally included (Figure 1). The present study was approved by the Institutional Review Board of the University of Navarra.

### 2.2. Assessment of Alcohol Consumption

Through a validated 136-item semiquantitative FFQ included in the baseline assessment, alcoholic beverage consumption (red wine, non-red wine, beer and spirits) was gathered. The baseline questionnaire also included further items on alcohol-drinking habits during the year preceding. Information including the maximum number of drinks consumed in a single weekday, or a day during the weekend, or in special occasions was collected in a categorical way (none, 1–2 drinks/day, 3–5 drinks/day, 6–9 drinks/day, 10–14 drinks/day and ≥15 drinks/day). For our analysis, binge drinking was considered as the consumption of at least 6 alcoholic drinks in a single day. This definition is motivated by the categories of the questionnaire, and the implications of using this definition are discussed in depth in the discussion section.

### 2.3. Breast Cancer Assessment

The diagnosis of breast cancer was initially self-reported. Patients who had reported a diagnosis of breast cancer were asked to provide a copy of their medical reports. These medical reports were subsequently used to confirm the cases by an independent expert who was blinded to the exposure. We also included as confirmed breast cancer cases, deaths due to breast cancer that had been identified through consultation of the National Death Index (NDI) in any case that the NDI adjudicated breast cancer as the cause of death. Alternatively, we included both confirmed and self-reported breast cancer cases pending confirmation as ‘probable breast cancer cases’.

### 2.4. Covariate Assessment

Information about sociodemographic, lifestyle and medical variables was obtained from the baseline questionnaire. The questionnaire included information on age of menopause, age of menarche, obstetric history, family history of breast cancer, and baseline chronic diseases.

Physical activity was assessed through a validated questionnaire [17]. Adherence to the Mediterranean dietary pattern was evaluated using the 9-item Mediterranean Diet Score defined by Trichopoulou et al. [18], a widely used index, but we removed the item of moderate alcohol consumption and used only the other 8 items.

Age at menopause was updated in the questionnaire after 16 years of follow-up. For those women with no available information on age at menopause, we used as cutoff point the 75th percentile of the age of menopause (52 years in our sample) [19].

### 2.5. Statistical Analysis

Quantitative baseline characteristics of participants were summarized with means and standard deviations, and qualitative traits with proportions across different binge drinking habits. To assess the relationship between binge drinking and the risk of breast cancer, we fitted Cox regression models. We estimated Hazard ratios (HR) and their 95% confidence intervals (CI) for women in the binge group compared to women reporting no binge drinking habits (reference category). We used age as underlying time variable and stratified our analyses by recruitment period and age (decades). We first fitted models for overall breast cancer incidence and then separated analyses according to menopausal status. Participants were followed up until the date of breast cancer diagnosis for both confirmed and probable cases, date of death or last contact, whichever occurred first. We adjusted a multivariable model including as potential confounders height (cm), family history of breast cancer (none, before, or after the age of 45 years), smoking habit (never, current, or former smoker), lifetime tobacco exposure (pack-years), age of menarche (<10, 10–16, >16 years), menopausal status, obstetric history (age < 25 years and nulliparous, age ≥ 25 years and nulliparous, first pregnancy before 25 years, first pregnancy between 25 and 30 years of age, first pregnancy being 30 years old or older), lifetime breast-feeding (months), hormone replacement therapy, years of university studies, physical activity (MET-h/week), alcohol consumption (g/day), adherence to the traditional Mediterranean Diet Score (0–8 points), total energy intake (kcal/day), body-mass index (BMI) (kg/m^2^), consumption of sugar-sweetened beverages (servings/day) and TV-watching (h/d). The consumption of sugar-sweetened beverages has been associated with breast cancer risk [20]. TV-watching was included in the model as a surrogate of sedentary behavior (also linked to breast cancer risk) [21]. As sensitivity analysis, we repeated our analyses for probable breast cancer cases and we included participants classified as abstainers at the baseline FFQ questionnaire.

Analyses were performed using STATA/SE version 15.0 (StataCorp), we used two-sided *p*-values and the statistical significance threshold was set a priori at 0.05.

## 3. Results

For the analysis, 9577 women were included, with a median follow-up of 11.8 years. Baseline characteristics of participants are described in Table 1 according to the binge drinking habit. In our cohort, 2292 participants (23.9%) were classified as binge drinkers at baseline. Women included in this group were younger (29.8 vs. 36 years), they had a higher mean alcohol intake (9.7 vs. 6.5 g/d), a higher proportion of nulliparity (82% vs. 62.7%) and a lower proportion of first pregnancy before the age of 30 years (8.6% vs. 22%) compared to non-binge drinkers. We also found differences in smoking habits, with a higher proportion of ever smokers (both current and former smokers) in the binge drinking group (62.5% vs. 44.5%) and a slightly higher lifetime tobacco exposure (4.7 vs. 4 pack-years). Both groups were rather similar at baseline in BMI, physical activity, adherence to Mediterranean Diet Score, and family history of breast cancer.

During a total follow-up of 104,932 women-years, 167 probable incident cases of breast cancer were identified, out of which 88 were confirmed. Women in the binge drinking group showed a higher risk of breast cancer (HR = 1.76; 95% CI: 1.03–2.99) compared to women in the non-binge drinking reference group, after adjusting for potential confounders (Table 2). According to menopausal status, the binge drinking pattern significantly increased the risk of premenopausal breast cancer (HR = 2.06; 95% CI: 1.11–3.82) when compared to non-binge drinking women. We found no significant associations for postmenopausal breast cancer. For each 10 g/d of alcohol consumed, we observed a 3% relative increase in the risk of breast cancer, although this association was not statistically significant (HR = 1.03; 95% CI: 0.78–1.36).

**Figure 2:**
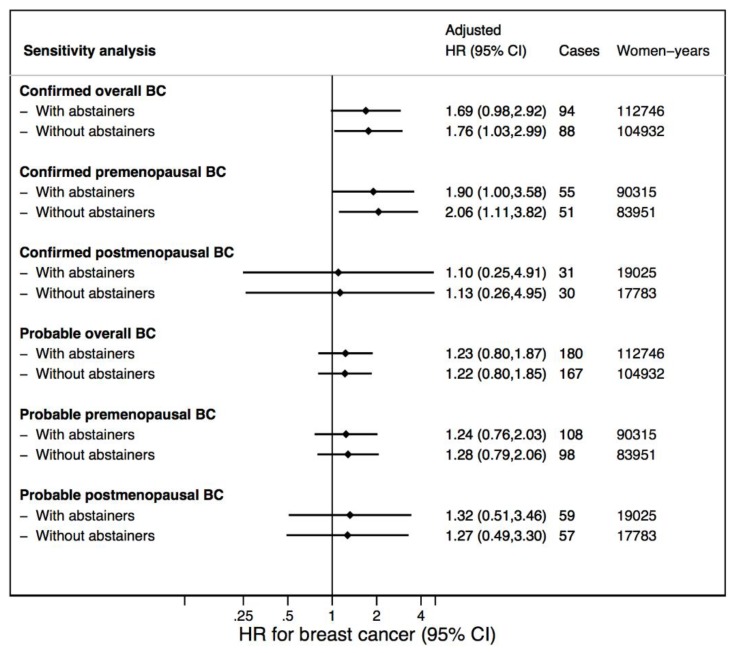
Sensitivity analyses showing Hazard Ratio (95% CI) of breast cancer—overall and stratified for menopausal status—with and without abstainers.

When we included probable breast cancer cases, results were attenuated (Table 2 and Table 3). The HR for breast cancer changed from 1.76 to 1.22 when we included as cases also the probable breast cancer cases. For probable premenopausal breast cancer, the HR decreased from 2.06 to 1.28 (losing statistical significance). Further sensitivity analyses are shown in Figure 2. When we included abstainers in the analysis, results barely changed. The HR for breast cancer changed from 1.76 to 1.69 when we included abstainers (for confirmed overall cases). For confirmed premenopausal breast cancer, the HR diminished from 2.06 to 1.90 when abstainers were included.

## 4. Discussion

In this prospective Mediterranean cohort, in relative terms, the risk of incident breast cancer was up to 76% higher in women with a binge drinking habit compared to women who drank without a binge drinking pattern.

Alcohol consumption has been linked to a higher risk of breast cancer [22,23,24,25,26]. Overall, a consistent association has been observed between alcohol intake and breast cancer among both pre- and post-menopausal women, and a linear dose-response association is widely accepted [27]. However, in the available evidence, the exposure to alcohol consumption has been mostly considered as grams/day and, contrarily, the pattern in which alcohol is consumed has not been directly addressed so frequently. In our study, we adjusted for the total amount of alcohol consumed in grams/day to appraise the association between the binge drinking pattern and the incidence of breast cancer. When we evaluated the risk associated with each 10 g increase of daily alcohol consumption, the estimated relative risk increase was 3%. This is slightly lower than the previously reported associated risk [9] and may be due to a higher adherence to a Mediterranean alcohol-drinking pattern [6]. As shown in Table 1, participants who consumed alcohol with a binge drinking pattern had a higher average alcohol intake than those who avoided binge drinking (mean difference 3.2 g/d, p < 0.001). These differences motivated the use of alcohol intake as a covariate in the model, to adjust for the effect of the quantity of consumed alcohol. Doing so, we assessed the association of the drinking pattern with the incidence of breast cancer within the same average alcohol intake.

Mediterranean Diet has been hypothesized to reduce the risk of breast cancer [28]. Assessment of the adherence to Mediterranean Diet Score (MDS) designed by Trichopoulou et al. has been widely used [18]. In this MDS, alcohol intake is considered in two categories (moderate vs. else). As the relationship between alcohol intake and breast cancer in the literature is linear, we considered this approach suboptimal to control for the potential confounding effect due to average alcohol intake (g/d). Therefore, we adjusted for the total amount of consumed alcohol in g/d and excluded the alcohol consumption from the MDS.

We identified three previous prospective cohorts that addressed the association between episodic heavy drinking and breast cancer risk. In the Danish Nurse Cohort study, women reporting binge drinking at weekends had a relative risk for breast cancer of 1.49 (95% CI 1.04–2.13) for 10–15 drinks and a relative risk of 2.51 (95% CI 1.37–4.59) for 16–21 drinks as compared to women reporting 1–3 drinks over the weekend [11]. In the Nurses’ Health Study, breast cancer risk was relatively increased by 51% (95% CI 1.35–1.70) in adult binge drinkers compared with abstainers, after controlling for cumulative alcohol consumption [9]. The most recently published study assessing the association between binge drinking and breast cancer risk is the Sister Study [12]. This prospective cohort enrolled 50,884 women residents of the United States or Puerto Rico with a family history of a sister with breast cancer. HR for breast cancer was 1.29 (95% CI 1.15–1.45) for ever binge drinking, compared to low-level drinking (<60 drinks/year). When compared with low-level drinkers who never binged, moderate drinkers (60–229 drinks/year) who binged showed a HR of 1.25 (95% CI 1.08–1.44). Authors found evidence of interaction between moderate lifetime drinking and binging. Thus, our results are consistent with the direct association between binge drinking and breast cancer risk found in previous studies, although the definition of binge was not consistent across studies.

In the stratified analysis, we found a 2-fold higher risk of premenopausal breast cancer associated with binge drinking compared to women who did not binge in our cohort. The estimated risk was attenuated when considering all the probable breast cancer diagnoses. This could be partially explained by the fact that some of the probable breast cancer cases were in fact cases of benign breast disease (e.g., fibroadenoma). Few epidemiologic studies have assessed the association between alcohol consumption and risk for benign breast disease and results have been so far inconclusive [29,30].

We found no association between binge drinking and postmenopausal breast cancer. This may be partially explained by our very low number of postmenopausal breast cancer cases, and a lower proportion of binge drinkers among postmenopausal women. Given the relatively young mean age of the SUN Project (34 years, SD = 10 years), we found a higher proportion of premenopausal breast cancer cases than postmenopausal cases. The scarcity of postmenopausal breast cancer cases limits our ability to obtain firm conclusions of differential associations between binge drinking and an early onset of breast cancer. Another potential explanation for the lack of association is not focusing on the etiologically relevant exposure period. Some authors suggest that alcohol consumption during adolescence and early adulthood may have a greater adverse effect on risk of breast disease, especially before the age of first pregnancy [31].

Multiple mechanisms have been described to explain the influence of alcohol in breast cancer risk. Alcohol per se can increase sex hormone levels and subsequently stimulate proliferation of estrogen-receptor positive cells [32]. The available evidence is consistent for a causal association between alcohol consumption and hormone receptor-positive tumors (otherwise luminal type) with inconclusive or no association with hormone receptor-negative tumors [33,34]. It has been suggested that the enzymatic machinery responsible for the elimination process of alcohol (alcohol dehydrogenase and aldehyde dehydrogenase) may not be sufficient during periods of binge drinking [35]. This can result in accumulation of carcinogenic products and reactive oxygen species, thus altering the DNA’s stability [36]. Binge drinking has also been associated with a higher risk of metabolic syndrome [37]. This can subsequently alter concentrations of many circulating hormones such as insulin, insulin-like growth factors (IGF), oestrogens, multiple adipokines and growth factors, creating an environment that may encourage breast carcinogenesis [14].

However, some limitations must be taken into consideration. First, the SUN Project participants are relatively young, which may partially explain the low incidence of breast cancer, especially of postmenopausal breast cancer. As a consequence, this may limit the statistical power. Nevertheless, the identified age-adjusted incidence was consistent with the reported incidence of breast cancer in the Spanish population [38]. Second, the self-reported exposure is a potential limitation as participants may tend to misreport their binge-drinking status. On the one hand, some binge-drinkers may have reported no binge-drinking in the past due to social desirability bias. On the other hand, as the question about binge inquired a period of one day instead of 2 h, some participants classified in the binge-drinking group may not actually be binge-drinkers. As a result of these potential biases, the observed association may be underestimated. Given the categorical assessment of binge-drinking habit used in the baseline questionnaire, we considered that the cut-off of ≥6 drinks/d would represent the binge-drinking habit more accurately in our cohort, although the usual definition is 4 drinks/2 h. Third, the exposure period considered may not be the most etiologically relevant for postmenopausal women, as drinking patterns may differ from the early adulthood. On the other hand, it seems to be an adequate window of exposure for premenopausal women, considering that for the vast majority of women we assessed alcohol consumption even before their first pregnancy. Fourth, the self-reported nature of the outcome assessment might have led to an underreporting of incident cases and thus to a lower sensitivity. Nevertheless, breast cancer cases were blindly confirmed—with high specificity—by an oncologist. Fifth, information on socio-economic status is not available and only the years of university studies could be included in the multivariable analysis. However, given that our sample was restricted to university graduates, it is fairly homogeneous in this aspect, a fact which may reduce the potential confounding effect of educational and socioeconomic status. Finally, the history of use of oral contraceptives was not included in the multivariate assessment because this information was not available in the baseline questionnaire.

To our best knowledge, this is the first study to assess the relationship between binge drinking and breast cancer risk in a middle-aged Mediterranean population. The prospective nature of the SUN Project ensures the temporal sequence between exposure and outcome, including a large sample size with a long follow-up and a good retention rate. Moreover, the adjustment for a wide number of potential confounders and the sensitivity analyses assure the robustness of our findings. Self-reported cancer cases were confirmed via medical reports to ensure that the final diagnosis was an invasive breast carcinoma and not a benign tumor such as lipoma or fibroadenoma. Lastly, by excluding abstainers in our analysis we aimed to compare the direct effect of binge drinking versus drinking in a manner different from the binge pattern. Previous studies have frequently used the abstainer group as the reference category. However, artificially elevated rates of disease in abstainers can be due to a higher morbidity among former drinkers or to the avoidance of alcohol drinking because of medical causes. This interpretation corresponds to the ‘sick quitter’ hypothesis, that may introduce some degree of bias [39] and by excluding abstainers we tried to avoid it.

## 5. Conclusions

In summary, in this Mediterranean cohort, a binge drinking habit was directly associated with a higher incidence of breast cancer. The risk of premenopausal breast cancer markedly increased for women in the binge drinking category. This study adds new evidence to the available knowledge concerning the adverse effects of heavy alcohol consumption on health.

## Figures and Tables

**Table 1:** Baseline characteristics of female participants in the SUN Project, according to binge drinking habit.

Variable	No Binge Drinking	Binge Drinking
*n* (%) *	7285 (76.1)	2292 (23.9)
Age (years)	36 (10.6)	29.8 (7.8)
Body-mass index (kg/m^2^)	22.3 (3.0)	22 (2.9)
Height (cm)	163 (6)	165 (5.9)
Physical activity (MET-h/week)	20 (18.5)	20.9 (19.7)
Total energy intake (kcal/day)	2303 (574)	2304 (563)
Alcohol intake (g/day)	6.5 (10.7)	9.7 (12.7)
Sugar-sweetened beverages (servings/day)	0.16 (0.33)	0.22 (0.32)
Lifetime breast-feeding (months)	3.1 (5.5)	1.2 (3.2)
Adherence to Mediterranean diet score	4.1 (1.7)	4.1 (1.7)
Time of university education (years)	4.8 (1.4)	4.7 (1.2)
Age of menarche (%)		
<10 years	20.3	19.4
10–16 years	71.7	72.5
>16 years	8	8.1
Menopause (%)	12.8	2.9
Obstetric history (including age at first pregnancy) (%)		
Age < 25 years & nulliparous	15.2	28.2
Age ≥ 25 years & nulliparous	47.5	53.8
First pregnancy before 25 years	5.3	1.6
First pregnancy between 25 & 30 years of age	16.7	7
First pregnancy being 30 years old or older	15.3	9.4
Time of hormone-replacement therapy (months)	1.2 (2.3)	1.6 (2.4)
Smoking (%)		
Never	55.5	37.5
Current	23	43.5
Former	21.5	19
Lifetime tobacco exposure (pack-years)	4 (7.7)	4.7 (7.2)
Family history of Breast Cancer (%)		
None	89.2	90.2
Before the age of 45 years	8.8	8.2
After the age of 45 years	2	1.6

* Values represent means (standard deviations), unless otherwise stated.

**Table 2:** Hazard ratio (95% CI) of overall breast cancer—confirmed cases—and by menopausal status according to binge drinking habit.

Confirmed Breast Cancer Cases	No Binge Drinking	Binge Drinking
**Overall**		
Cases/women-years	67/80,452	21/24,479
Age adjusted	1 (Ref.)	1.82 (1.09–3.03)
Multivar. adjusted *	1 (Ref.)	1.76 (1.03–2.99)
**Premenopausal**		
Cases/women-years	33/61,155	18/22,796
Age adjusted	1 (Ref.)	2.29 (1.27–4.13)
Multivar. adjusted *	1 (Ref.)	2.06 (1.11–3.82)
**Postmenopausal**		
Cases/women-years	28/16,405	2/1377
Age adjusted	1 (Ref.)	0.84 (0.20–3.53)
Multivar. adjusted *	1 (Ref.)	1.13 (0.26–4.95)

* Adjusted for height (cm), family history of breast cancer (no history, before 45 years, after 45 years), smoking habit (never, former or current smoker), lifetime tobacco exposure (pack-years), age at menarche (<10 years, 10–16 years or >16 years), obstetric history (5 categories), lifetime breast-feeding (months), years of university studies (years), Mediterranean Diet Score (0–8), alcohol consumption (g/d), total daily energy intake (tertiles of kcal/d), body-mass index (kg/m^2^), consumption of sugar-sweetened beverages (servings/day) and TV-watching (h/d). Only for postmenopausal women: hormone replacement therapy (yes/no), duration of hormone replacement therapy (months) and age at menopause (<50 years, 50–55 years or >55 years). Age as underlying time variable. Stratified analyses by recruitment period and age (decades).

**Table 3:** Hazard ratio (95% CI) of overall breast cancer—probable cases—and by menopausal status according to binge drinking habit.

Probable Breast Cancer Cases	No Binge Drinking	Binge Drinking
**Overall**		
Cases/women-years	135/80,452	32/24,479
Age adjusted	1 (Ref.)	1.29 (0.87–1.94)
Multivar. adjusted *	1 (Ref.)	1.22 (0.81–1.85)
**Premenopausal**		
Cases/women-years	72/61,155	26/22,796
Age adjusted	1 (Ref.)	1.45 (0.92–2.30)
Multivar. adjusted *	1 (Ref.)	1.28 (0.79–2.06)
**Postmenopausal**		
Cases/women-years	52/16,405	5/1377
Age adjusted	1 (Ref.)	1.10 (0.44–2.79)
Multivar. adjusted *	1 (Ref.)	1.27 (0.49–3.30)

* Adjusted for height (cm), family history of breast cancer (no history, before 45 years, after 45 years), smoking habit (never, former or current smoker), lifetime tobacco exposure (pack-years), age at menarche (<10 years, 10–16 years or >16 years), obstetric history (5 categories), lifetime breast-feeding (months), years of university studies (years), Mediterranean Diet Score (0–8), alcohol consumption (g/d), total daily energy intake (tertiles of kcal/d), body-mass index (kg/m^2^), consumption of sugar-sweetened beverages (servings/day) and TV-watching (h/d). Only for postmenopausal women: hormone replacement therapy (yes/no), duration of hormone replacement therapy (months) and age at menopause (<50 years, 50–55 years or >55 years). Age as underlying time variable. Stratified analyses by recruitment period and age (decades).
